# Career Adaptability and Academic Achievement Among Chinese High School Students: A Three-Wave Longitudinal Study of Social Cognitive and Metacognitive Mediating Mechanisms

**DOI:** 10.3390/jintelligence14060111

**Published:** 2026-06-18

**Authors:** Ziluo Yan, Zhiyu Xu, Le Zhang, Yutong Guo

**Affiliations:** 1 School of Education, Nanjing University, Nanjing 210023, China; ziluoyan@nju.edu.cn (Z.Y.); 602024120001@smail.nju.edu.cn (Y.G.); 2 School of Education Science, Hunan Normal University, Changsha 410081, China; 3 Academic Affairs Office, Xi’an Academy of Fine Arts, Xi’an 710065, China; 4 Faculty of Education, Beijing Normal University, Beijing 100875, China; 5 Xinjiang Academy of Educational Sciences, Urumqi 830049, China

**Keywords:** career adaptability, academic achievement, academic self-efficacy, academic outcome expectations, metacognitive strategies

## Abstract

Career adaptability has been linked to academic achievement, yet the mechanisms underlying this association remain insufficiently understood, particularly among adolescents in highly competitive, exam-oriented educational systems. Based on Career Construction Theory (CCT) and the performance model of Social Cognitive Career Theory (SCCT), this study tested whether academic self-efficacy, academic outcome expectations, and metacognitive strategies mediated this association. A three-wave longitudinal study was conducted with 519 students from two general high schools in central China (40.85% boys; mean age = 16.28 years, SD = 0.82). Career adaptability was measured at Time 1, the three mediators were measured at Time 2, and academic achievement was measured at Time 1 and Time 3 using standardized examination scores in Chinese, mathematics, and English. After controlling for baseline achievement and demographic covariates, structural equation modeling with bias-corrected bootstrapping showed that T1 career adaptability had a significant total effect on T3 academic achievement, whereas the direct effect was nonsignificant after the mediators were included. Significant indirect effects were found through academic self-efficacy and metacognitive strategies. Academic outcome expectations did not significantly mediate the association, and the pathway from academic self-efficacy to academic outcome expectations was not supported. These findings indicate that career adaptability may contribute to later academic achievement mainly through students’ academic self-efficacy and metacognitive strategy use.

## 1. Introduction

As high school students face the dual demands of academic learning and career exploration (D. Brown, 2002), career adaptability serves as a crucial psychosocial resource that helps them clarify future goals, manage educational transitions, and cope with increasingly uncertain career pathways (Savickas & Porfeli, 2012). As a core adaptability resource, career adaptability may matter not only for occupational but also for academic outcomes (Negru-Subtirica & Pop, 2016). This is because, in contemporary society, prolonged participation in formal education and increasing social uncertainty have made future career pursuits more closely intertwined with present academic goals (Heckhausen & Tomasik, 2002; Negru, 2012; Negru et al., 2013). Accordingly, adolescents with stronger career adaptability may view school learning as more closely connected to future educational and career opportunities, thereby sustaining greater academic engagement and performance (Negru-Subtirica & Pop, 2016).

Empirical studies have generally supported this link, showing positive associations between career adaptability and academic achievement (Akkermans et al., 2018; Negru-Subtirica & Pop, 2016; N. Wang et al., 2024). However, the psychological mechanisms underlying this relationship remain insufficiently understood, particularly in the Chinese high school context, where academic learning and career preparation are closely intertwined under the Gaokao system. As China’s National College Entrance Examination, the Gaokao represents a high-stakes selection mechanism that shapes students’ access to higher education and is widely recognized as one of the most consequential competitions in Chinese students’ educational lives (Cheng & Hamid, 2025; Zhao et al., 2024).

To address this gap, this study used a three-wave longitudinal design to examine whether academic self-efficacy, academic outcome expectations, and metacognitive strategies mediated the association between career adaptability and later academic achievement among high school students in central China. By integrating CCT and the performance model of SCCT, the study aimed to clarify how future-oriented career resources may be linked to school performance through cognitive and learning-regulation mechanisms. This study may inform school-based career education and academic support by identifying specific psychological and strategic learning factors that can be strengthened in high-stakes examination contexts.

## 2. Literature Review

### 2.1. Theoretical Framework

Career Construction Theory (CCT) provides a useful lens for explaining why career adaptability may be relevant to students’ academic development. CCT views career development as an adaptive process in which individuals use psychosocial resources to respond to changing developmental, educational, and work-related demands (Savickas, 2005; Savickas & Porfeli, 2012). In this model, career adaptability is located at the resource level and refers to individuals’ capacity to prepare for future tasks, make choices, explore possible pathways, and cope with uncertainty. These resources are especially salient in adolescence, when students begin to make educational choices that are closely tied to later career opportunities (Negru-Subtirica & Pop, 2016). In the present study, academic achievement is treated as an educational adaptation outcome because it reflects how students respond to school demands and invest in learning tasks (Poropat, 2009). CCT also suggests that adaptability resources are translated into outcomes through more concrete responses to developmental demands. In the academic domain, metacognitive strategies may be understood as learning-related regulatory responses through which students plan, monitor, evaluate, and adjust their learning when facing academic challenges (Ku & Ho, 2010; Pintrich, 2004; Zimmerman, 2002). Thus, while metacognitive strategies originate from the metacognition and self-regulated learning literature, they are consistent with the CCT logic that adaptability resources are expressed through context-specific responses before being reflected in adaptation outcomes.

The performance model of Social Cognitive Career Theory (SCCT) further explains how career adaptability may be linked to academic achievement through cognitive mechanisms. According to this model, ability and prior performance are associated with later academic or occupational performance both directly and indirectly through social-cognitive variables, especially self-efficacy beliefs, outcome expectations, and performance goals (S. D. Brown et al., 2008; Lent et al., 1994; Lent & Brown, 2019). Self-efficacy shapes how individuals use their abilities by influencing effort, persistence, and goal-directed behavior, whereas outcome expectations contribute to performance by shaping the goals individuals set and pursue (Bandura, 1986; Lent & Brown, 2019). Career adaptability can be understood as a meta-competency, or a higher-order capacity to learn and adapt across situations rather than to adjust to a single task (Hall & Chandler, 2005). Therefore, the resources it provides may generalize to the academic domain. Applied to the present study, students with higher career adaptability may be more likely to view current education as relevant to future career development, which may strengthen their academic self-efficacy and academic outcome expectations (Avram et al., 2019; Zeng et al., 2022). Academic self-efficacy and academic outcome expectations were therefore examined as cognitive mechanisms linking career adaptability to academic achievement.

### 2.2. Career Adaptability and Academic Achievement

Career adaptability is a set of psychosocial self-regulatory, transactional, and malleable resources that allow individuals to prepare for, cope with, and manage career transitions and the associated career- or work-related issues (Savickas, 1997, 2013). It is usually represented by four dimensions—concern, control, curiosity, and confidence—which respectively capture future orientation, agency in decision making, exploration of self and environment, and perceived capacity to handle career-related tasks (Savickas, 2013). For adolescents, career construction begins during the school years (Negru-Subtirica & Pop, 2016; Roeser et al., 2000). Those with higher career adaptability are more likely to invest effort in academic tasks, as school achievement represents an important foundation for future career development, which may in turn contribute to better academic performance (Avram et al., 2019). Academic achievement is a multi-dimensional concept, usually measured by the academic performance of teenagers at school, more specifically their grade point average (GPA) (Poropat, 2009). In the context of Chinese education, examination results remain the most commonly used measurement indicator (Phillipson & Phillipson, 2007). Influenced by the college entrance examination selection mechanism and the cultural tradition of emphasizing educational achievements, academic achievement has long been a major concern for families, schools, and society (Q. Wang & Pomerantz, 2009).

According to CCT, career adaptability can be viewed as an adaptability resource that may be associated with adaptation outcomes, including academic achievement in the context of schooling (Poropat, 2009; Savickas & Porfeli, 2012). This association has been supported by both cross-sectional and longitudinal evidence. Avram et al. (2019) reported that Romanian undergraduates with higher career adaptability tended to have higher GPA, while Negru-Subtirica and Pop (2016) found reciprocal links between career concern and academic achievement among adolescents aged 13 to 19. Research in the Chinese context has likewise shown that high school students with different latent profiles of career adaptability differ significantly in their subsequent academic achievement (N. Wang et al., 2024). However, although the association between career adaptability and academic achievement has been documented in both cross-sectional and longitudinal work, the mechanisms linking career adaptability to later academic performance remain less clear. Prior research has tended to focus on whether career adaptability is related to achievement, while giving less attention to the cognitive and learning-regulation processes through which this association may occur.

### 2.3. Academic Self-Efficacy, Academic Outcome Expectations, and Social Cognitive Career Theory

Academic self-efficacy refers to students’ beliefs in their capability to learn and perform academic tasks successfully (Artino, 2012; Bandura, 1997; Chemers et al., 2001; Schunk & DiBenedetto, 2020). Prior studies have shown that career adaptability is positively associated with academic self-efficacy, possibly because career-adaptable students are more likely to approach academic challenges with planning, confidence, and future-oriented purpose (Yi et al., 2025; Zeng et al., 2022). In turn, empirical and meta-analytic evidence has consistently linked academic self-efficacy to academic achievement (Alivernini & Lucidi, 2011; Honicke & Broadbent, 2016; Robbins et al., 2004). Taken together, these findings suggest that academic self-efficacy may be a plausible cognitive mechanism through which career adaptability is associated with academic achievement, although evidence for the career adaptability–self-efficacy link remains less extensive and has often relied on cross-sectional designs.

Academic outcome expectations refer to students’ beliefs about the likely academic consequences of their learning engagement and performance (Bandura, 1997; Lent et al., 1994; Springer et al., 2001). In this study, academic outcome expectations refer specifically to students’ beliefs that their current academic efforts and performance will lead to desirable academic outcomes. Prior research indicates that positive outcome expectations are associated with students’ goal setting, learning engagement, persistence, and academic achievement (Cupani & Pautassi, 2013; Lent & Brown, 2019; Sheu et al., 2018). Career adaptability may also support academic outcome expectations because more career-adaptable adolescents tend to be future-oriented and better able to connect present academic efforts with anticipated educational and vocational outcomes (Ginevra et al., 2016; Jia et al., 2022; Negru-Subtirica & Pop, 2016; Xu et al., 2023). Thus, academic outcome expectations may represent another cognitive pathway linking career adaptability to academic achievement. However, because prior evidence has more often examined career- or development-related outcome expectations than academic-oriented outcome expectations, direct longitudinal evidence for this specific academic pathway remains limited.

Academic self-efficacy and academic outcome expectations are also theoretically related. Students who believe they can succeed academically may be more likely to expect positive outcomes from their academic efforts (Bandura, 1997; Lent et al., 1994; Lent & Brown, 2019). Accordingly, the present study specified a pathway from academic self-efficacy to academic outcome expectations. However, because both constructs were measured at T2, this pathway should be interpreted as theoretically specified rather than as evidence of temporal precedence between the two mediators.

### 2.4. Metacognitive Strategies and Career Construction Theory

Metacognitive strategies are regulatory learning strategies that enable students to supervise and control their thinking and learning processes (Ku & Ho, 2010). They include planning, monitoring, evaluating, and adjusting strategies during learning (Veenman & Elshout, 1999). As a core component of self-regulated learning, metacognitive strategies capture how students regulate their learning processes, rather than merely whether they hold positive academic beliefs (Pintrich, 2004; Zimmerman, 2002).

Career adaptability may be associated with students’ use of metacognitive strategies. Students with stronger career adaptability tend to be more future-oriented, more proactive, and more capable of managing developmental challenges (Savickas, 2013; Savickas & Porfeli, 2012). Related research has shown that career adaptability is associated with career planning, exploration, occupational self-efficacy, career decision-making self-efficacy, engagement, future orientation, proactive career behaviors, self-regulation, and academic engagement (Ginevra et al., 2016; Merino-Tejedor et al., 2016; Rudolph et al., 2017; Taber & Blankemeyer, 2015). Taken together, these findings suggest that career-adaptable students may be more likely to approach learning in a deliberate and self-regulated manner. However, direct evidence linking career adaptability to metacognitive strategy use remains limited.

Evidence for the link between metacognitive strategies and academic achievement is stronger. Prior meta-analyses have shown that metacognition is positively associated with academic achievement broadly (He et al., 2024), with particularly strong effects in mathematics (Xie et al., 2024). Intervention meta-analyses have also found that metacognitive strategy instruction and broader learning strategy instruction improve students’ academic performance (de Boer et al., 2018; Donker et al., 2014). Thus, metacognitive strategies may function as a learning-regulation pathway linking career adaptability to academic achievement.

### 2.5. Conceptual Distinctions Among the Key Constructs

Because several constructs in the model concern students’ capacity to manage academic or developmental demands, we clarified their conceptual boundaries before hypothesis testing. Career adaptability, academic self-efficacy, academic outcome expectations, and metacognitive strategies differ in theoretical origin, level of specificity, and functional role. These distinctions are summarized in Table 1.

### 2.6. Current Study

The present study aims to examine the longitudinal relationship between career adaptability and academic achievement among high school students in central China, as well as its underlying psychological mechanisms. Although prior research has documented a positive association between career adaptability and academic achievement (Akkermans et al., 2018; Negru-Subtirica & Pop, 2016; N. Wang et al., 2024), three gaps remain. First, most existing studies have relied on cross-sectional designs (Akkermans et al., 2018; Avram et al., 2019), which limit clarification of temporal ordering among predictors, mediators, and outcomes. Second, few studies have integrated CCT and SCCT to simultaneously examine cognitive and behavioral mediating pathways. Third, limited attention has been paid to Chinese high school students, whose academic pressures and career decisions are deeply intertwined under the Gaokao system (Zhao et al., 2024), suggesting that the underlying mechanisms may exhibit context-specific features.

**Figure 1:**
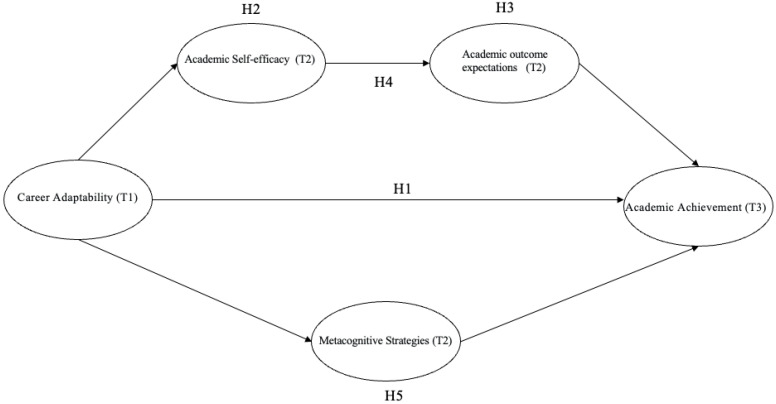
Hypothesized longitudinal mediation model. Note. T1 = Time 1; T2 = Time 2; T3 = Time 3. Solid arrows represent hypothesized paths. Sex, age, and prior academic achievement at T1 were controlled but are not displayed for clarity.

To address these gaps, the present study adopted a three-wave prospective longitudinal design, drawing on CCT as the overarching framework and the performance model of SCCT as the explanatory framework for cognitive mechanisms. Specifically, we examined whether T1 career adaptability was associated with T3 academic achievement at the level of the total effect and tested whether this association operated through T2 academic self-efficacy, T2 academic outcome expectations, and T2 metacognitive strategies. The hypothesized longitudinal mediation model is presented in Figure 1.

First, CCT suggests that adaptability resources facilitate adaptation outcomes (Savickas & Porfeli, 2012), and prior studies have shown a positive association between career adaptability and academic achievement (N. Wang et al., 2024). Accordingly, we expected T1 career adaptability to be positively associated with T3 academic achievement at the level of the total effect. Because career adaptability is a relatively distal developmental resource, this association may operate primarily through more proximal cognitive and behavioral mechanisms. We proposed the following hypothesis:

**H1.**  *T1 career adaptability is positively associated with T3 academic achievement at the total-effect level.*

Second, the performance model of SCCT posits that self-efficacy and outcome expectations serve as key cognitive mechanisms linking personal resources to academic performance (Lent et al., 1994; Lent & Brown, 2019). Prior research has shown that career adaptability is positively associated with academic self-efficacy (Yi et al., 2025), and that self-efficacy and outcome expectations are related to academic performance (Lent & Brown, 2019). Accordingly, we proposed the following indirect-pathway hypotheses:

**H2.**  *T1 career adaptability is indirectly associated with T3 academic achievement through T2 academic self-efficacy.*

**H3.**  *T1 career adaptability is indirectly associated with T3 academic achievement through T2 academic outcome expectations.*

**H4.**  *T1 career adaptability is indirectly associated with T3 academic achievement through the theoretically specified pathway linking T2 academic self-efficacy and T2 academic outcome expectations.*

Third, CCT proposes that adaptability resources are translated into adaptation outcomes through concrete adapting responses (Savickas, 2013). Metacognitive strategies, which involve executive learning behaviors such as planning, monitoring, and adjustment, can be conceptualized as a typical form of adapting response in the academic domain (Ku & Ho, 2010). Prior research has shown that career adaptability is positively associated with proactive planning and self-regulation (Ginevra et al., 2016), and that metacognitive strategies contribute to academic achievement (He et al., 2024). Accordingly, we proposed:

**H5.**  *T1 career adaptability is indirectly associated with T3 academic achievement through T2 metacognitive strategies.*

## 3. Materials and Methods

### 3.1. Participants

The data used in this study were drawn from a large-scale longitudinal research project on the career development of adolescents and university students. Students were recruited from two general public academic high schools in Henan Province, central China. The schools were selected purposively based on their substantive relevance to the research questions and their capacity to support longitudinal school-based data collection. Both schools operated within the Gaokao-oriented educational system and represented ordinary academic high school settings in the local context. This made them appropriate settings for examining how career adaptability is associated with academic achievement among students who face simultaneous demands of subject learning, academic competition, and future educational planning. The selection of the schools was also shaped by design considerations. The three-wave longitudinal design required schools with relatively stable student cohorts, comparable examination arrangements, and institutional support for repeated assessments. The two schools used the same midterm and final examination papers during the study period and were able to provide comparable examination records across waves. This helped ensure the comparability of the academic achievement measure across the two schools. Within each school, intact classes were recruited using cluster sampling to reduce disruption to regular teaching activities and to maintain the integrity of classroom-based data collection.

Across the three waves, 721 valid responses were obtained at T1, 609 were retained at T2, and 519 were retained at T3. This indicated that 112 participants were lost from T1 to T2 (attrition rate = 15.53%) and that 90 additional participants were lost from T2 to T3 (attrition rate = 14.78%). The overall retention rate relative to T1 was 71.98%. Only students who completed all three waves were included in the final analytic sample to preserve the intended T1–T2–T3 temporal structure of the longitudinal mediation model. FIML was used to handle item-level missing data within this complete-wave analytic sample, but it was not used to retain participants who dropped out across waves.

The final analytic sample consisted of 519 valid participants, including 212 boys (40.85%) and 307 girls (59.15%). Among them, 275 were enrolled in Grade 10 (52.99%) and 244 in Grade 11 (47.01%). Because Grade 10 students had not yet undergone academic track selection, subject-track classification was recorded only for Grade 11 students, among whom 97 were enrolled in the humanities track (39.75%) and 147 in the science track (60.25%). Participants ranged in age from 14.17 to 17.92 years, with a mean age of 16.28 years (SD = 0.82). Detailed demographic characteristics of the samples across the three waves are presented in Table 2.

### 3.2. Participant Attrition

Following prior longitudinal studies (e.g., Dou et al., 2025; R. Zhang et al., 2023), we conducted attrition analyses by comparing completers and dropouts on demographic characteristics and key study variables (see Table 3). Among the 721 participants who took part at T1, 519 completed all three waves and were classified as the complete group, whereas the remaining 202 participants who did not complete all waves were classified as the dropout group. For T1 variables, because all participants completed the baseline assessment, comparisons were made between the complete group (*n* = 519) and all dropouts (*n* = 202). For T2 variables, because only participants who completed the second wave had available T2 data, comparisons were conducted between the complete group (*n* = 519) and those who participated at T2 but were lost at T3 (*n* = 90). Minimal group differences and small effect sizes would suggest a low risk of serious systematic bias due to attrition, indicating that the data were broadly consistent with the Missing at Random (MAR) assumption. Missing item-level data within the final analytic sample were handled using FIML.

The results indicated that, for demographic variables, there were no significant differences between the complete and dropout groups in terms of sex, χ^2^(1) = 0.004, *p* = .953, φ = 0.002, or grade, χ^2^(1) = 0.015, *p* = .902, φ = 0.005. For T1 study variables, no significant differences were found between the two groups in career adaptability, t(719) = 0.07, *p* = .941, d = 0.006, or academic achievement, t(719) = 0.93, *p* = .354, d = 0.08. Regarding T2 variables, a significant difference was observed only for academic outcome expectations, t(607) = 1.99, *p* = .047, d = 0.23, although the effect size was small. No significant differences were found for academic self-efficacy, t(607) = 0.04, *p* = .966, d = 0.01, or metacognitive strategies, t(607) = 1.32, *p* = .187, d = 0.15. Overall, most variables did not differ significantly between completers and dropouts, and the observed effect sizes were generally small. These results suggest that attrition did not appear to introduce substantial systematic bias, although potential attrition-related bias cannot be fully ruled out.

### 3.3. Procedure

To test the hypothesized mediation effects, this study used a three-wave longitudinal design (Kline, 2023), in which key variables were measured at three distinct time points. This design helped reduce the risk of common method bias (Podsakoff et al., 2003) and provided a clearer temporal ordering among the predictor, mediators, and outcome variables. As Maxwell and Cole (2007) noted, mediation reflects a temporal process, and cross-sectional data are limited in establishing such temporal sequence. Accordingly, career adaptability was measured at T1, the proposed mediators at T2, and academic achievement at T3, which provided a stronger basis for examining the hypothesized longitudinal mediation model, although causal inferences should still be made cautiously. This design has also been widely adopted in prior studies testing longitudinal mediation models (e.g., Qu et al., 2021; R. Zhang et al., 2023).

All surveys were administered by trained research assistants in classroom settings. T1 was conducted in October 2024, around the autumn-semester midterm examination. At this wave, students completed measures of demographic information and career adaptability, and their academic achievement records were collected from homeroom teachers with students’ consent. T2 was conducted in January 2025, around the autumn-semester final examination. At this wave, students completed measures of academic self-efficacy, academic outcome expectations, and metacognitive strategies. T3 was conducted in May 2025, around the spring semester midterm examination. At this wave, academic achievement records were collected in the same manner as at T1. The two participating schools used identical examination papers at the corresponding assessment points, supporting the comparability of academic achievement across schools.

### 3.4. Measures

All study variables were measured using established instruments that have been widely applied in prior research or adapted from validated scales. In the present study, all measures demonstrated acceptable psychometric properties. The reliability and validity results for each scale are presented in Table 4 and Table 5.

For measures that required adaptation to the present academic and cultural context, we followed a multi-step adaptation procedure. First, the original items were reviewed by the research team to ensure conceptual consistency with the target construct. Second, items were translated into Chinese or revised into academic-context wording where necessary. Third, two researchers with expertise in educational psychology and career development reviewed the adapted items for semantic clarity, age appropriateness, and contextual relevance to Chinese high school students. Disagreements were discussed until consensus was reached. Finally, the psychometric properties of the adapted scales were evaluated in the present sample through reliability analysis and confirmatory factor analysis.

#### 3.4.1. Career Adaptability Scale at Wave 1

Career adaptability was measured with the Chinese form of the Career Adapt-Abilities Scale (Hou et al., 2012). This instrument assesses four adaptability resources: concern, control, curiosity, and confidence. Each subscale contains six items, yielding 24 items in total. Students rated the extent to which they had developed each ability on a 5-point scale ranging from 1 (not strong) to 5 (strongest). Example items include “thinking about what my future will be like” for concern, “making decisions by myself” for control, “exploring my surroundings” for curiosity, and “taking care to do things well” for confidence. Subscale scores were calculated by averaging the corresponding items, and higher scores indicated stronger career adaptability.

#### 3.4.2. Academic Self-Efficacy at Wave 2

Academic self-efficacy was assessed using an adapted version of the General Self-Efficacy Scale (Schwarzer et al., 1997). Because the original scale assesses generalized self-efficacy, the items were revised to fit the academic context while retaining the original scale structure and core meaning. For example, general references to coping with difficulties or handling unexpected situations were modified to refer specifically to learning tasks, academic challenges, and study-related goals. The adapted items were reviewed by two researchers with expertise in educational psychology and career development to ensure semantic clarity, developmental appropriateness, and consistency with the construct of academic self-efficacy. The scale consists of 10 items rated on a 4-point Likert scale ranging from 1 (not at all true) to 4 (exactly true). Sample items include “For me, it is easy to persist in my academic aspirations and achieve my learning goals” and “With my abilities, I can handle unexpected situations in my studies.” Item scores were summed, with higher total scores indicating higher levels of academic self-efficacy. In the present study, the adapted scale showed acceptable reliability and construct validity. Before constructing the latent variable in the SEM, we examined the ten academic self-efficacy items at the item level. The scale showed acceptable internal consistency, with Cronbach’s α = 0.810. Given the theoretical unidimensionality of academic self-efficacy, three parcels were used to reduce item-specific measurement noise and model complexity (Bandalos, 2002; Little et al., 2002; Matsunaga, 2008). Parcel scores were computed by averaging prespecified item sets.

#### 3.4.3. Academic Outcome Expectations at Wave 2

Academic outcome expectations were assessed using the academic-oriented dimension of the Educational Outcome Expectations Scale developed by Şeker and Çapri (2022). Because the original scale was developed in a different cultural and educational context, the items were translated and adapted for Chinese high school students. The translation aimed to preserve the meaning of the original items while ensuring that the wording was understandable and relevant in the Chinese academic context. The adapted items were reviewed by two researchers with expertise in educational psychology and career development, who evaluated whether the items adequately captured students’ expectations that current learning engagement and academic performance would lead to desirable academic outcomes. Minor wording adjustments were made to improve contextual relevance, particularly in relation to university entrance examinations. This dimension consists of four items rated on a 5-point Likert scale ranging from 1 (strongly disagree) to 5 (strongly agree). Sample items include “With the education I have received and will receive, I will be able to prepare adequately for university entrance exams” and “With the education I have received and will receive, I will be able to get a result I want in the university entrance exams.” Higher scores indicate stronger academic outcome expectations. In the present study, the adapted scale showed acceptable reliability and construct validity.

#### 3.4.4. Metacognitive Strategies at Wave 2

Metacognitive strategies were assessed using the metacognitive strategies subscale of the Self-Regulated Learning Strategies Questionnaire for secondary school students, adapted by Kong and Lu (2012) from the Motivated Strategies for Learning Questionnaire (MSLQ). The subscale consists of six items measuring students’ use of metacognitive strategies in planning, monitoring, and regulating their learning processes. All items were rated on a 5-point Likert scale ranging from 1 (strongly disagree) to 5 (strongly agree). Sample items include “I ask myself questions to make sure I understand what I have been studying” and “I try to change my way of learning to adapt to the teacher’s teaching style and classroom management requirements.” Higher total scores indicate greater use of metacognitive strategies.

#### 3.4.5. Academic Achievement at Wave 1 and Wave 3

Academic achievement was measured using students’ school examination scores in three core subjects: Chinese, mathematics, and English. Scores were collected at T1 and T3, with T1 representing the autumn-semester midterm examination and T3 representing the spring-semester midterm examination. Each subject had the same maximum score. For each student, the raw scores in Chinese, mathematics, and English were first summed to obtain a total examination score, so the three subjects contributed equally to the composite score. The total score was then standardized within grade at each wave and converted into a z-score. Higher z-scores indicated higher relative academic achievement within the same grade and wave. The T1 and T3 examinations were not identical in content because they were administered in different semesters. However, both were formal midterm examinations covering the corresponding semester curriculum in the same three core subjects and followed comparable school assessment arrangements. Therefore, the standardized achievement scores should be interpreted as students’ relative academic standing within grade and wave.

### 3.5. Covariates

In all analyses, sex (1 = male, 2 = female), age, grade (1 = Grade 10, 2 = Grade 11), and Time 1 academic achievement were statistically controlled, because prior studies have indicated that these background variables may be associated with the main constructs examined in this study (Akkermans et al., 2018; Zacher, 2014).

### 3.6. Statistical Analysis

Data analysis proceeded in seven steps. First, data screening and preprocessing—including removal of invalid questionnaires, identification of multivariate outliers, and examination of skewness and kurtosis—were conducted in SPSS 26.0, followed by attrition analyses comparing retained and dropout participants on demographic characteristics and core study variables. Second, the missing data mechanism was assessed and full information maximum likelihood (FIML) estimation was applied to handle missing data. Third, descriptive statistics and Pearson correlations were computed for all focal variables. Fourth, item analysis and reliability testing were performed for all questionnaire-based measures. Steps 2–4 were also conducted in SPSS 26.0. Fifth, confirmatory factor analysis (CFA) was conducted in Mplus 8.3 (Muthén & Muthén, 2017) to examine the measurement model, with reliability and validity evaluated using standardized factor loadings, composite reliability (CR), average variance extracted (AVE), and discriminant validity indices. Model fit was evaluated using a consistent set of commonly recommended fit indices, including χ^2^, df, χ^2^/df, CFI, TLI, RMSEA, and SRMR. Sixth, the longitudinal mediation model was estimated in Mplus 8.3 to test the direct and indirect effects of T1 career adaptability on T3 academic achievement through T2 academic self-efficacy, academic outcome expectations, and metacognitive strategies, controlling for T1 academic achievement and demographic covariates. Finally, bias-corrected bootstrapping with 5000 resamples (Preacher & Hayes, 2008) was used to test the significance of indirect effects, with mediation inferred when the 95% confidence interval excluded 0.

## 4. Results

### 4.1. Common Method Bias

Given that several variables in this study were measured using students’ self-report questionnaires, common method bias (CMB) was taken into consideration (Podsakoff et al., 2003). To reduce its potential influence, several procedural measures were adopted. Specifically, the questionnaire items were arranged carefully, different constructs were presented in separate sections with clear instructions, and participants were assured of the anonymity and confidentiality of their responses. In addition, the items were worded in a neutral and straightforward manner. More importantly, this study used a three-wave longitudinal design, with the independent variable, mediators, and outcome variable measured at different time points. Academic achievement was also obtained from school examination records rather than students’ self-reports. These design features helped reduce the likelihood of common method bias.

To further examine this issue, Harman’s single-factor test was conducted. The first unrotated factor accounted for 20.02% of the total variance, which was below the commonly used 40% cutoff. A confirmatory factor analysis was also performed by specifying a single-factor model in which all self-reported items loaded onto one latent factor. The model fit was poor (χ^2^ = 975.65, df = 119, χ^2^/df = 8.20, CFI = 0.67, TLI = 0.62, RMSEA = 0.12, SRMR = 0.10). Taken together, these results suggest that common method bias was not a serious concern in the present study.

### 4.2. Assessment of Reliability and Validity

Before testing the structural model, the psychometric properties of the four self-report measures were examined, including T1 career adaptability, T2 academic self-efficacy, T2 academic outcome expectations, and T2 metacognitive strategies (see Table 4 and Table 5). The Cronbach’s alpha coefficients were 0.87, 0.81, 0.74, and 0.78, respectively, all above the recommended cutoff of 0.70, indicating acceptable internal consistency (Kline, 2023). As shown in Table 4, all standardized factor loadings exceeded 0.50. Composite reliability (CR) values ranged from 0.74 to 0.85, also meeting the recommended criterion of 0.70. The average variance extracted (AVE) values ranged from 0.38 to 0.65. The AVE values for career adaptability (0.43), academic outcome expectations (0.42), and metacognitive strategies (0.38) were below the commonly recommended threshold of 0.50, indicating that convergent validity for these constructs was weaker than ideal. However, their composite reliability values were 0.75, 0.74, and 0.78, respectively, all above the 0.70 criterion. Following Fornell and Larcker (1981), AVE values below 0.50 may still be considered tolerable when composite reliability is adequate. Moreover, all standardized factor loadings exceeded 0.50 and were in the expected direction, and removing indicators would have reduced construct coverage and comparability with prior research. The HTMT values ranged from 0.17 to 0.68, all below 0.90, supporting discriminant validity (Henseler et al., 2015). Overall, the measures showed acceptable reliability and discriminant validity, although convergent validity for several constructs was weaker than ideal.

### 4.3. Descriptive Statistics and Correlation Analysis

A correlation analysis was conducted among the core variables in this study. As shown in Table 6, T1 career adaptability was positively correlated with T2 academic self-efficacy (r = 0.46, *p* < .001), T2 academic outcome expectations (r = 0.27, *p* < .001), T2 metacognitive strategies (r = 0.42, *p* < .001), and T3 academic achievement (r = 0.36, *p* < .001). T2 academic self-efficacy was also positively correlated with T2 academic outcome expectations (r = 0.13, *p* < .01), T2 metacognitive strategies (r = 0.56, *p* < .001), and T3 academic achievement (r = 0.49, *p* < .001). In addition, T2 academic outcome expectations were positively associated with T2 metacognitive strategies (r = 0.12, *p* < .01) and T3 academic achievement (r = 0.13, *p* < .01), whereas T2 metacognitive strategies showed a significant positive correlation with T3 academic achievement (r = 0.53, *p* < .001). Among these associations, the strongest correlation was observed between metacognitive strategies and academic achievement. Regarding the covariates, sex was not significantly associated with any of the focal variables. Age showed only a weak positive correlation with metacognitive strategies. Baseline academic achievement at T1 was positively associated with T3 academic achievement and metacognitive strategies, but was not significantly related to career adaptability, academic self-efficacy, or academic outcome expectations.

### 4.4. Longitudinal Mediation Analysis

To examine the mechanism through which T1 career adaptability predicted T3 academic achievement, a structural equation model was estimated in Mplus 8.3 following the bootstrap mediation procedure recommended by Preacher and Hayes (2008), while controlling for sex, age, grade, and T1 academic achievement. In the model, the pathway linking academic self-efficacy and outcome expectations was specified on theoretical grounds, whereas metacognitive strategies served as a parallel mediator. The model showed a good fit to the data (χ^2^ = 281.72, df = 173, χ^2^/df = 1.63, CFI = 0.96, TLI = 0.95, RMSEA = 0.04, SRMR = 0.05). The path analysis showed that T1 career adaptability positively predicted T2 academic self-efficacy (β = 0.68, *p* < .001, C.R. = 9.93), T2 outcome expectations (β = 0.46, *p* < .001, C.R. = 4.40), and T2 metacognitive strategies (β = 0.66, *p* < .001, C.R. = 9.13). However, the path from T2 academic self-efficacy to T2 outcome expectations was not significant (β = −0.15, *p* = .10, C.R. = −1.64). At the outcome level, both T2 metacognitive strategies (β = 0.30, *p* < .001, C.R. = 4.39) and T2 academic self-efficacy (β = 0.26, *p* < .001, C.R. = 4.25) positively predicted T3 academic achievement, whereas the effect of T2 outcome expectations on T3 academic achievement was not significant (β = 0.04, *p* = .34, C.R. = 0.95). After the mediators were included, the direct effect of T1 career adaptability on T3 academic achievement was no longer significant (β = 0.09, *p* = .32, C.R. = 1.00) (see Table 7).

To clarify the nonsignificant path of academic outcome expectations in the full model, supplementary diagnostic analyses were conducted. Academic outcome expectations were positively associated with T3 academic achievement (*r* = 0.132, *p* = .003), and multicollinearity diagnostics revealed no cause for concern (VIF = 1.089 for academic outcome expectations; maximum VIF = 1.629 across all predictors). Stepwise regression analyses (see Supplementary Table S1) further showed that the association between academic outcome expectations and T3 academic achievement was statistically significant when controlling for sex, age, grade, and T1 academic achievement (*β* = 0.152, *p* < .001), attenuated after academic self-efficacy was added (*β* = 0.092, *p* = .008), and became nonsignificant in the full observed-variable model incorporating academic self-efficacy, metacognitive strategies, and career adaptability (*β* = 0.051, *p* = .132). This pattern is more consistent with shared explanatory variance among the predictors than with severe multicollinearity or a suppression effect.

To further test the mediating effects, bias-corrected bootstrap analyses based on 5000 resamples were conducted (see Table 8). The results showed that the direct effect of T1 career adaptability on T3 academic achievement was not significant (DE = 0.329, 95% BC CI [−0.304, 0.970], *p* = .289). Regarding the specific indirect effects, the indirect effect through T2 academic self-efficacy was significant (IE1 = 0.623, 95% BC CI [0.358, 1.017], *p* < .001), and the indirect effect through T2 metacognitive strategies was also significant (IE4 = 0.679, 95% BC CI [0.377, 1.160], *p* < .001). In contrast, the indirect effect through T2 academic outcome expectations was not significant (IE2 = 0.066, 95% BC CI [−0.075, 0.247], *p* = .300), and the proposed indirect pathway involving T2 academic self-efficacy and T2 academic outcome expectations was likewise not significant (IE3 = −0.015, 95% BC CI [−0.097, 0.012], *p* = .227). The total indirect effect was significant (TIE = 1.353, 95% BC CI [0.889, 2.122], *p* < .001), and the total effect was also significant (TE = 1.682, 95% BC CI [1.286, 2.190], *p* < .001). Taken together, these findings suggest that T1 career adaptability was associated with T3 academic achievement primarily through T2 academic self-efficacy and T2 metacognitive strategies, whereas the mediating role of academic outcome expectations, either alone or in sequence with academic self-efficacy, was not supported.

**Figure 2:**
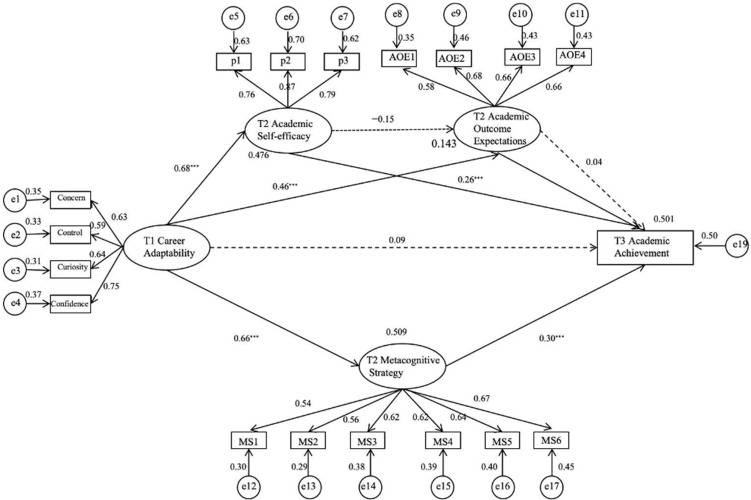
The Final Structural Model. Note. Standardized coefficients are presented. Solid lines indicate significant paths, whereas dashed lines indicate nonsignificant paths. Error terms are omittedfor clarity. T1 = Time 1; T2 = Time 2; T3 = Time 3. *** *p* < .001.

As shown in Figure 2, the R^2^ values indicated that the model explained 47.7% of the variance in T2 academic self-efficacy, 14.3% of the variance in T2 academic outcome expectations, and 50.9% of the variance in T2 metacognitive strategies. Meanwhile, the combined effects of T1 career adaptability, T2 academic self-efficacy, T2 academic outcome expectations, T2 metacognitive strategies, and the control variables accounted for 50.1% of the variance in T3 academic achievement. These findings suggest that the proposed model had satisfactory explanatory power for the endogenous variables, particularly for T3 academic achievement.

Taken together, these findings indicate that H1 was supported with respect to the total effect, although the direct effect of T1 career adaptability on T3 academic achievement was not significant after the mediators were included. Regarding the indirect pathways, H2 and H5 were supported, whereas H3 and H4 were not supported. Specifically, significant indirect effects were found through T2 academic self-efficacy and T2 metacognitive strategies, but not through T2 academic outcome expectations or the proposed pathway from T2 academic self-efficacy to T2 academic outcome expectations.

## 5. Discussion

Based on Career Construction Theory (Savickas, 2005, 2013) and Social Cognitive Career Theory (S. D. Brown & Lent, 2023; Lent & Brown, 2019), this three-wave prospective longitudinal study examined how career adaptability was associated with later academic achievement among high school students in central China. The findings showed that, after controlling for T1 academic achievement and demographic variables, T1 career adaptability had a significant total association with T3 academic achievement. However, its direct association with T3 academic achievement was no longer significant after the proposed mediators were included. Significant indirect pathways were found through T2 academic self-efficacy and T2 metacognitive strategies. In contrast, neither the indirect pathway through T2 academic outcome expectations nor the theoretically specified pathway linking T2 academic self-efficacy and T2 academic outcome expectations was supported. Taken together, these results suggest that career adaptability may be associated with later academic achievement primarily through more proximal cognitive and behavioral mechanisms, rather than through an independent direct pathway.

### 5.1. Career Adaptability as a Distal Resource for Academic Achievement

The finding indicates that career adaptability was positively associated with later academic achievement overall. Specifically, T1 career adaptability showed a significant total effect on T3 academic achievement, supporting H1 at the total-effect level. However, this association was no longer significant after academic self-efficacy, academic outcome expectations, and metacognitive strategies were included in the model. Thus, career adaptability should not be interpreted as exerting an independent direct effect on academic achievement. This pattern extends previous studies that reported positive direct or bivariate associations between career adaptability and academic achievement (Avram et al., 2019; Negru-Subtirica & Pop, 2016; N. Wang et al., 2024). Specifically, career adaptability may function as a relatively distal developmental resource whose academic value is realized through more proximal cognitive and behavioral mechanisms, particularly academic self-efficacy and metacognitive strategies.

This finding is consistent with CCT, which distinguishes among adaptability resources, adapting responses, and adaptation outcomes (Savickas, 2013; Savickas & Porfeli, 2012). Career adaptability provides students with future orientation, a sense of control, exploratory orientation, and confidence, corresponding to the four dimensions of concern, control, curiosity, and confidence. However, these psychosocial resources may not automatically translate into better academic achievement. The present findings suggest that career adaptability may provide students with developmental direction and motivational resources, but its academic value is more likely to emerge when these resources are transformed into academic self-efficacy and effective learning strategies.

This interpretation is particularly meaningful in the Chinese high school context. Under the Gaokao-oriented educational system, academic achievement is closely tied to students’ future educational and occupational opportunities (Cheng & Hamid, 2025; Yu et al., 2018). Families, schools, and the broader educational culture also tend to connect current academic learning with future developmental opportunities (Phillipson & Phillipson, 2007; Zhao et al., 2024). At the student level, however, having a future orientation or recognizing the importance of academic success may not be sufficient. Future-oriented beliefs may support motivation, but academic performance also depends on students’ confidence in handling academic tasks and their ability to regulate learning processes (Bandura, 1997; Lent & Brown, 2019; Pintrich, 2004; Zimmerman, 2002). Thus, career adaptability may become practically relevant to achievement when it is accompanied by stronger academic self-efficacy and more effective metacognitive regulation. This helps explain why the direct pathway from career adaptability to later academic achievement was not significant after the mediators were included.

### 5.2. The Mediating Role of Academic Self-Efficacy

The findings indicated academic self-efficacy significantly mediated the association between career adaptability and subsequent academic achievement, providing support for H2. This finding is consistent with prior evidence on the mechanism linking career adaptability, domain-specific self-efficacy, and adaptation outcomes. For example, Guan et al. (2013), in a three-wave study of Chinese university graduates’ job search process, found that career adaptability predicted job search outcomes through job search self-efficacy. Similarly, Duffy et al. (2015) showed that career adaptability was indirectly associated with academic satisfaction through career decision self-efficacy. Although these studies focused on job search outcomes and academic satisfaction rather than objective academic achievement, they suggest a common mechanism: career adaptability may be linked to positive career or academic adaptation outcomes through more specific domain-based efficacy beliefs. Extending this line of work, the present study shows that this mechanism also applies to academic achievement among high school students. Specifically, career adaptability may be linked not only to career-related outcomes or academic satisfaction through career-related self-efficacy, but also to later academic performance through academic self-efficacy.

This finding aligns with SCCT, which identifies self-efficacy as a central social-cognitive mechanism linking personal resources to performance-related outcomes (Lent et al., 1994; Lent & Brown, 2019). Students with higher career adaptability may possess stronger concern, control, curiosity, and confidence (Savickas, 2013). These general adaptive resources may help students develop more positive beliefs about their academic capabilities, including the belief that they can complete learning tasks, cope with academic difficulties, and achieve academic goals (Guan et al., 2013). Prior research also suggests that career exploration may strengthen students’ learning motivation and academic self-efficacy (Rogers et al., 2008). In addition, when students have a clearer understanding of their career interests and the practical meaning of learning, their autonomy, learning motivation, and self-efficacy may be enhanced (Jin, 2020). Higher academic self-efficacy may further contribute to academic achievement by encouraging students to set challenging goals, sustain effort, and persist when facing academic difficulties (Bandura, 1997). In contrast, students with lower self-efficacy are more likely to view difficult tasks as threats and give up easily (Guan et al., 2013). Thus, academic self-efficacy may help explain why career adaptability is associated with better academic performance over time. Therefore, academic self-efficacy can be understood as an important proximal social-cognitive mechanism linking career adaptability to later academic achievement (Zhao et al., 2024).

### 5.3. The Mediating Role of Metacognitive Strategies

The results showed that metacognitive strategies significantly mediated the association between career adaptability and subsequent academic achievement, supporting H5. This finding indicates that career adaptability is not merely a future-oriented developmental resource but may also be reflected in concrete regulatory behaviors in students’ current learning processes. This interpretation is consistent with CCT, which suggests that adaptability resources influence adaptation outcomes through specific adapting responses (Savickas, 2013; Savickas & Porfeli, 2012). In the present study, metacognitive strategies can be understood as such adapting responses in the academic domain.

This finding can be explained by integrating Career Construction Theory with self-regulated learning theory. First, from the perspective of CCT, career adaptability is viewed as a relatively malleable psychosocial resource closely related to self-regulation in managing developmental tasks and career transitions (Savickas, 2013; Savickas & Porfeli, 2012). Prior research has also shown that career adaptability is associated with adapting responses, such as career planning, career exploration, and proactive career behaviors (Johnston, 2018; Rudolph et al., 2017). These findings suggest that students with higher career adaptability may be more likely to actively manage their own developmental process. Extending this logic to the academic domain, metacognitive strategies may be understood as a concrete form of self-regulation in learning (Pintrich, 2004; Zimmerman, 2002). Students with higher career adaptability may be more likely to connect current learning with future development, and therefore to plan their learning, monitor their understanding, and adjust their learning strategies more actively.

Second, metacognitive strategies are themselves an important learning mechanism for academic achievement. Self-regulated learning theory suggests that successful learners do not passively complete learning tasks. Rather, they actively plan, monitor, and regulate their learning processes (Pintrich, 2004; Zimmerman, 2002). Metacognitive strategies help students clarify learning goals, check whether they truly understand learning materials, identify weaknesses in learning, and adjust their methods based on feedback. In this way, metacognitive strategies may support learning efficiency and help students use their learning effort more effectively. Meta-analytic evidence also shows that metacognition and metacognitive strategy instruction are positively associated with academic performance (de Boer et al., 2018; Donker et al., 2014; Ohtani & Hisasaka, 2018). Taken together, metacognitive strategies may serve as a key mediating mechanism because they connect the future orientation and active developmental awareness provided by career adaptability with the concrete learning-regulation behaviors required for academic achievement.

### 5.4. Implications

#### 5.4.1. Theoretical Implications

The present findings carry several theoretical implications. First, by integrating CCT and SCCT within a three-wave longitudinal design, this study provides a more process-oriented understanding of the link between career adaptability and academic achievement. Whereas previous research has mainly documented positive associations between career adaptability and academic outcomes (Avram et al., 2019; Negru-Subtirica & Pop, 2016), our findings suggest that career adaptability functions as a distal developmental resource whose academic value is realized through more proximal mechanisms, particularly academic self-efficacy and metacognitive strategies. This finding refines the CCT proposition that adaptability resources are linked to adaptation outcomes through adapting responses (Savickas, 2013; Savickas & Porfeli, 2012).

Second, the study contributes to SCCT by clarifying which cognitive mediators are most consequential for academic achievement in the high school stage. Although SCCT positions both self-efficacy and outcome expectations as core social-cognitive mediators (Lent & Brown, 2019), our results indicate that, once academic self-efficacy and metacognitive strategies are accounted for, academic outcome expectations did not show a significant unique mediating effect on later academic achievement. In addition, the theoretically specified pathway linking T2 academic self-efficacy and T2 academic outcome expectations was not supported. Because both mediators were measured at T2, this result should not be interpreted as evidence for or against a temporal ordering between academic self-efficacy and academic outcome expectations. Rather, it indicates that the proposed theory-based pathway did not yield a significant indirect effect in the present model. One plausible explanation is that academic outcome expectations may be relatively homogeneous under the highly standardized and exam-oriented Gaokao system. In this context, most adolescents share similar normative expectations about the value of academic success for college admission and future careers, which may reduce the explanatory power of between-person variation in outcome expectations (Cheng & Hamid, 2025; Zhao et al., 2024).

Third, by conceptualizing metacognitive strategies as an academic-domain adapting response, this study extends CCT into the learning domain and bridges career development theory with self-regulated learning research (Pintrich, 2004; Zimmerman, 2002). The findings suggest that career adaptability is not merely a future-oriented psychosocial resource but is also embodied in students’ day-to-day regulatory behaviors, providing a theoretical basis for connecting career construction with the science of learning.

#### 5.4.2. Practical Implications

The findings also offer practical implications. First, because career adaptability indirectly contributes to academic achievement through academic self-efficacy and metacognitive strategies, schools should avoid treating career education as a stand-alone activity separated from academic learning. Career education may be more useful when it helps students connect future goals with current subject learning. This recommendation is consistent with CCT, which views career adaptability as a psychosocial resource for managing developmental tasks and transitions (Savickas, 2013; Savickas & Porfeli, 2012). It is also consistent with evidence suggesting that career interventions may support adolescents’ career adaptability and career decision-making self-efficacy (N. Wang et al., 2024). In practice, career-planning activities may guide students to identify future educational or occupational goals, break them into short-term academic targets, and reflect on how current subject learning is related to future opportunities.

Second, schools should not rely only on strengthening students’ positive expectations about future academic outcomes. In a Gaokao-oriented context, many students may already understand that academic achievement is closely tied to future educational and career opportunities. However, the present findings suggest that such recognition may have limited practical value unless students also develop confidence in their ability to handle academic tasks. Therefore, teachers and school counselors should place greater emphasis on strengthening academic self-efficacy. Practical strategies may include helping students set attainable short-term learning goals, providing mastery-oriented feedback, guiding students to reflect on successful learning experiences, and supporting them in coping with temporary academic setbacks. These practices may help students transform future-oriented academic expectations into more concrete confidence in their capacity to learn and perform.

Third, the significant indirect pathway through metacognitive strategies suggests that schools may benefit from embedding metacognitive regulation into everyday teaching. This recommendation is consistent with self-regulated learning theory, which emphasizes planning, monitoring, and evaluation as core learning processes (Pintrich, 2004; Zimmerman, 2002). It is also supported by meta-analytic evidence linking metacognitive strategy instruction and broader learning strategy instruction with improved academic performance (de Boer et al., 2018; Donker et al., 2014). Teachers may ask students to make learning plans, monitor strategy use, keep brief reflection logs, and adjust study methods after tests or assignments.

### 5.5. Limitations and Future Directions

This study has several limitations. First, the sample was drawn from two general public academic high schools in Henan Province, central China. Both schools operated within the Gaokao-oriented educational system and represented ordinary academic high school settings in the local context. However, this regional school-based sample limits the generalizability of the findings. China’s regions differ substantially in socioeconomic conditions and educational opportunities, which may shape students’ career-related beliefs, academic attitudes, and access to school support, especially across urban and rural settings or across coastal and inland areas (J. Zhang et al., 2015). School type, school academic ranking, and local Gaokao pressure may also influence the mechanisms examined in this study. Therefore, the present findings should be viewed as evidence from two general public high schools in central China rather than as representative of all Chinese high school students. Future studies should include students from more provinces, school types, urban and rural areas, and schools with different academic rankings.

Second, several key constructs were measured using student self-report questionnaires, including career adaptability, academic self-efficacy, academic outcome expectations, and metacognitive strategies. Although academic achievement was obtained from school examination records, the self-reported constructs may still be influenced by social desirability, response style, and students’ subjective understanding of their own learning processes. In addition, the intervals between the three waves may not fully capture the developmental processes linking career adaptability, academic beliefs, metacognitive strategies, and academic achievement. Because all mediators were measured at T2, temporal separation was established between T1 career adaptability, the T2 mediators, and T3 academic achievement, but not among the mediators themselves. Future studies could combine self-report scales with teacher ratings, behavioral indicators, learning analytics, or interview data, and use longer follow-up periods with more measurement waves to test these processes more rigorously.

Finally, the present model focused on intrapersonal cognitive and behavioral mediators. Career development and academic achievement are also shaped by contextual factors such as parental involvement, teacher support, peer influence, and socioeconomic status (Akkermans et al., 2018; Zacher, 2014). Future research could integrate these contextual variables as moderators or additional mediators to develop a more ecologically valid model of how career adaptability is associated with academic achievement.

## 6. Conclusions

This study examined the longitudinal association between career adaptability and academic achievement among high school students in central China. It also tested the mediating roles of academic self-efficacy, academic outcome expectations, and metacognitive strategies. Three main findings should be highlighted. First, T1 career adaptability had a significant total association with T3 academic achievement, but this association was primarily indirect rather than direct. Second, academic self-efficacy and metacognitive strategies represented the main significant indirect pathways linking career adaptability to later academic achievement. Third, academic outcome expectations and the theoretically specified pathway linking academic self-efficacy and academic outcome expectations did not show significant indirect effects.

Taken together, these findings suggest that career adaptability should not be understood as an independent direct academic advantage; rather, it may function as a distal developmental resource whose academic relevance appears to be linked mainly to more proximal cognitive and behavioral mechanisms. By integrating Career Construction Theory and Social Cognitive Career Theory, this study provides preliminary longitudinal evidence on the indirect associations between adolescents’ career development resources and academic achievement in a central Chinese high school context. Its relevance lies in shifting attention from whether career adaptability is associated with achievement to how this association may operate through academic self-efficacy and metacognitive regulation. These findings also suggest that career education in similar high school contexts may benefit from being connected with academic self-efficacy enhancement and metacognitive strategy training.

## Figures and Tables

**Table 1:** Conceptual and Theoretical Distinctions Among the Key Constructs.

Construct	Theoretical Foundation	Core Meaning in This Study	Distinction from Related Constructs
Career adaptability	Career Construction Theory; career construction model of adaptation (Savickas, 2005; Savickas & Porfeli, 2012; Hirschi et al., 2015; Rudolph et al., 2017)	Career-development-oriented psychosocial resources that help individuals cope with career-related developmental tasks, educational transitions, and future uncertainty.	Differs from generalized self-regulatory competence because it is specifically oriented toward career-related and educational transitions rather than general goal regulation across domains. It also differs from adapting responses because it represents adaptability resources rather than situated coping behaviors.
Confidence	Career Construction Theory; Career Adapt-Abilities Scale (Savickas, 2005; Savickas & Porfeli, 2012)	Positive beliefs about one’s ability to solve problems, overcome obstacles, and manage future career-related challenges.	Differs from academic self-efficacy because it is broader and more future-oriented. It concerns career-related problem solving and transitions rather than specific academic task performance.
Academic self-efficacy	Social Cognitive Theory; Social Cognitive Career Theory (Bandura, 1997; Lent et al., 1994)	Students’ perceived capability to organize and execute actions required for successful academic performance.	Differs from career adaptability confidence because it is more domain- and task-specific. It also differs from metacognitive strategies because it concerns capability beliefs rather than strategy use.
Academic outcome expectations	Social Cognitive Career Theory (Lent et al., 1994)	Students’ beliefs about the likely academic benefits or consequences of engaging in learning-related behaviors.	Differs from academic self-efficacy because it concerns expected consequences rather than perceived capability. In SCCT terms, self-efficacy concerns “Can I do this?”, whereas outcome expectations concern “What will happen if I do this?”
Metacognitive strategies	Metacognition and self-regulated learning theory (Flavell, 1979; Pintrich, 2004; Zimmerman, 2002)	Students’ use of planning, monitoring, regulating, and evaluating strategies during learning.	Differs from academic self-efficacy because it reflects strategy use rather than capability beliefs. Differs from career adaptability because it concerns regulation of current learning processes rather than broader readiness for future career-related transitions.

**Table 2:** Sample Characteristics Across Three Measurement Waves.

Characteristic	T1 (*n* = 721)	T2 (*n* = 609)	T3 (*n* = 519)
Sex			
Male	294 (40.78%)	258 (42.36%)	212 (40.85%)
Female	427 (59.22%)	351 (57.64%)	307 (59.15%)
Grade			
Grade 10 (Senior 1)	381 (52.84%)	323 (53.04%)	275 (52.99%)
Grade 11 (Senior 2)	340 (47.16%)	286 (46.96%)	244 (47.01%)
Academic track (Grade 11 only) ^a^			
Humanities	—	—	97 (39.75%)
Sciences	—	—	147 (60.25%)

Note. T1 = Time 1 (October 2024); T2 = Time 2 (January 2025); T3 = Time 3 (May 2025). Age was computed as of December 2024 using birth year and month. ^a^ Academic track (humanities vs. sciences) applies only to Grade 11 students; Grade 10 students had not yet been assigned to a track at T1 or T2. Track percentages are based on Grade 11 students with valid track data in the T3 complete sample (*n* = 244).

**Table 3:** Attrition Analysis of Demographic Characteristics and Study Variables.

Variable	Complete Group (*n* = 519) M (SD) or %	Dropout Group (*n* = 202) M (SD) or %	Test Statistic	*p*	Effect Size
Demographic variables (chi-square tests)					
Sex (% male)	212 (40.85%)	83 (41.09%)	χ^2^(1) = 0.004	0.953	φ = 0.002
Grade (% Grade 10)	275 (52.99%)	106 (52.48%)	χ^2^(1) = 0.015	0.901	φ = 0.005
T1 study variables (independent-samples *t* tests)					
Career adaptability	3.54 (0.48)	3.54 (0.47)	t(719) = 0.07	0.941	d = 0.006
Academic achievement (z)	0.04 (0.97)	−0.04 (0.90)	t(719) = 0.93	0.354	d = 0.08
T2 study variables: complete group vs. T3 dropout only (*n* = 90)					
Academic self-efficacy	2.39 (0.47)	2.39 (0.45)	t(607) = 0.04	0.966	d = 0.01
Academic outcome expectations	3.57 (0.56)	3.44 (0.53)	t(607) = 1.99	0.047 *	d = 0.23
Metacognitive strategies	3.51 (0.60)	3.42 (0.58)	t(607) = 1.32	0.19	d = 0.15

Note. Complete group = participants who completed all three waves of data collection (*n* = 519). For demographic variables and T1 study variables, the dropout group refers to all participants who did not remain in the study through T3 (*n* = 202). For T2 study variables, the dropout group includes only participants who provided data at T2 but were lost at T3 (*n* = 90). Participants who dropped out before T2 were not included in these comparisons because T2 data were unavailable for them. * *p* < .05.

**Table 4:** Psychometric Properties of the Study Measures.

Construct	Indicator	M (SD)	Loading	Kurtosis	Skewness	α	Model Fit
Career Adaptability	Concern	3.30 (0.69)	0.63	−0.14	0.14	0.87	χ^2^ = 530.23, df = 241, χ^2^/df = 2.20, CFI = 0.91, TLI = 0.90, RMSEA = 0.04, SRMR = 0.05
	Curiosity	3.57 (0.62)	0.59	−0.28	0.18		
	Control	3.88 (0.62)	0.64	−0.44	0.13		
	Confidence	3.40 (0.59)	0.75	0.09	−0.34		
Academic Self-Efficacy	Parcel 1	2.21 (0.54)	0.76	−0.24	−0.15	0.81	Three-parcel one-factor model was just-identified.
	Parcel 2	2.56 (0.54)	0.87	0.17	0.19		
	Parcel 3	2.45 (0.55)	0.79	1.15	1.33		
Academic Outcome Expectations	AOE1	3.83 (0.75)	0.58	−0.24	−0.07	0.74	χ^2^ = 0.56, df = 2, χ^2^/df = 0.28, CFI = 1.00, TLI = 1.00, RMSEA = 0.00, SRMR = 0.01
	AOE2	3.68 (0.85)	0.68	−0.13	−0.45		
	AOE3	3.86 (0.79)	0.66	−0.25	−0.31		
	AOE4	3.71 (0.79)	0.66	−0.05	−0.52		
Metacognitive Strategies	MS1	3.55 (0.86)	0.54	−0.11	−0.27	0.78	χ^2^ = 10.62, df = 9, χ^2^/df = 1.18, CFI = 0.98, TLI = 0.99, RMSEA = 0.02, SRMR = 0.01
	MS2	3.37 (0.88)	0.56	−0.15	−0.15		
	MS3	3.66 (0.82)	0.62	−0.15	−0.49		
	MS4	3.49 (0.91)	0.62	−0.17	−0.34		
	MS5	3.37 (0.86)	0.64	−0.04	−0.29		
	MS6	3.61 (0.86)	0.67	−0.06	−0.48		

Note. Standardized factor loadings are reported. χ^2^ = chi-square; CFI = comparative fit index; TLI = Tucker–Lewis index; RMSEA = root mean square error of approximation; SRMR = standardized root mean square residual.

**Table 5:** Convergent and discriminant validity of the study constructs.

Construct	AVE	CR	HTMT			
			1	2	3	4
1. Career Adaptability	0.43	0.75	0.65			
2. Academic Self-Efficacy	0.65	0.85	0.57	0.81		
3. Academic Outcome Expectations	0.42	0.74	0.38	0.17	0.65	
4. Metacognitive Strategies	0.38	0.78	0.56	0.68	0.17	0.62

Note. AVE = average variance extracted; CR = composite reliability; HTMT = heterotrait–monotrait ratio of correlations. Values on the diagonal are the square roots of AVE, and values below the diagonal are HTMT coefficients.

**Table 6:** Means, Standard Deviations, and Correlations among Study Variables.

Variable	M	SD	1	2	3	4	5
1. Career Adaptability (T1)	3.54	0.47	—				
2. Academic Self-Efficacy (T2)	2.39	0.47	0.46 ***	—			
3. Academic Outcome Expectations (T2)	3.77	0.6	0.27 ***	0.13 **	—		
4. Metacognitive Strategies (T2)	3.51	0.6	0.42 ***	0.56 ***	0.12 **	—	
5. Academic Achievement (T3)	0	1	0.36 ***	0.49 ***	0.13 **	0.53 ***	—
Covariates							
6. Sex	—	—	−0.03	−0.07	−0.03	−0.08	0.03
7. Age	16.28	0.82	0.08	0.05	0	0.10 *	0.03
8. Grade	1.47	0.50	0.12 **	0.06	−0.06	0.13 **	0.16 ***
9. Academic Achievement (T1)	0.04	0.97	0.01	0.07	−0.04	0.22 ***	0.44 ***

Note. * *p* < .05, ** *p* < .01, *** *p* < .001.

**Table 7:** Structural path coefficients of the longitudinal mediation model.

Panel/Path	β	C.R.	*p*
Panel A. Predicting T2 mediators			
T1 Career Adaptability → T2 Academic Self-Efficacy	0.68	9.93	<.001
T1 Career Adaptability → T2 Academic Outcome Expectations	0.46	4.4	<.001
T1 Career Adaptability → T2 Metacognitive Strategies	0.66	9.13	<.001
T2 Academic Self-Efficacy → T2 Academic Outcome Expectations	−0.15	−1.64	.10
Panel B. Predicting T3 academic achievement			
T2 Academic Self-Efficacy → T3 Academic Achievement	0.26	4.25	<.001
T2 Academic outcome Expectations → T3 Academic Achievement	0.04	0.95	.34
T2 Metacognitive Strategies → T3 Academic Achievement	0.3	4.39	<.001
T1 Career Adaptability → T3 Academic Achievement	0.09	1.00	.32

Note. Sex, age, grade, and T1 academic achievement were included as covariates in the model. β = standardized path coefficient; C.R. = critical ratio.

**Table 8:** Direct, indirect, and total effects of T1 career adaptability on T3 academic achievement.

Path	Effect Estimate	SE	95% BC CI	*p*
Direct Effect				
T1 career adaptability → T3 academic achievement	0.329	0.323	[−0.304, 0.970]	0.289
Indirect Effect				
T1 career adaptability → T2 academic self-efficacy → T3 academic achievement	0.623	0.165	[0.358, 1.017]	<.001
T1 career adaptability → T2 academic outcome expectations → T3 academic achievement	0.066	0.078	[−0.075, 0.247]	0.300
T1 career adaptability → T2 academic self-efficacy → T2 academic outcome expectations → T3 academic achievement	−0.015	0.024	[−0.097, 0.012]	0.227
T1 career adaptability → T2 metacognitive strategies → T3 academic achievement	0.679	0.195	[0.377, 1.160]	<.001
Total indirect effect	1.353	0.302	[0.889, 2.122]	<.001
Total effect	1.682	0.233	[1.286, 2.190]	<.001

Note. SE = bootstrap standard error; BC CI = bias-corrected confidence interval. Indirect effects were estimated using bootstrap resampling with 5000 samples. An effect was considered statistically significant when the 95% BC CI did not include zero.

## Data Availability

The data presented in this study are not publicly available due to privacy and ethical restrictions. The data are available from the corresponding author upon reasonable request.

## Supplementary Materials

The following supporting information can be downloaded at: https://www.mdpi.com/article/10.3390/jintelligence14060111/s1, Table S1: Regression Analyses Examining Academic Outcome Expectations.

## Abbreviations

The following abbreviations are used in this manuscript:

CCT	Career Construction Theory
SCCT	Social Cognitive Career Theory
T1/T2/T3	Time 1/Time 2/Time 3
FIML	Full Information Maximum Likelihood
CFA	Confirmatory Factor Analysis
CMB	Common Method Bias
AVE	Average Variance Extracted
CR	Composite Reliability
HTMT	Heterotrait–Monotrait Ratio
