# Supplementary material for: Career Adaptability and Academic Achievement Among Chinese High School Students: A Three-Wave Longitudinal Study of Social Cognitive and Metacognitive Mediating Mechanisms
Source: J Intell. 2026 Jun 18;14(6):111. doi: 10.3390/jintelligence14060111 (PMC13302216; doi:10.3390/jintelligence14060111)
Supplement: Supplementary file 1 [file jintelligence-14-00111-s001.zip › jintelligence-4320099-supplementary.pdf]

Supplementary Materials

Supplementary Table S1. Regression analyses examining academic outcome expectations

Model	Unstandardized *B*	*SE*	Standardized *β*	*t*	*p*	Model Fit R^2	*Adj.* R^2	ΔR^2	*F*	*p*
Model 1	—	—	—	—	—	.192	.186	.192	30.543	< .001
Model 2	0.256	0.066	.152	3.883	< .001	.215	.207	.023	15.079	< .001
Model 3	0.154	0.057	.092	2.684	.008	.415	.408	.199	174.438	< .001
Model 4	0.085	0.056	.051	1.510	.132	.472	.464	.057	27.630	< .001

Note. The dependent variable was T3 academic achievement. B, SE, β, t, and p refer to the coefficient of academic outcome expectations. Model 1 included sex, age, grade, and T1 academic achievement. Model 2 added academic outcome expectations. Model 3 additionally added academic self-efficacy. Model 4 additionally added metacognitive strategies and career adaptability. β = standardized regression coefficient; SE = standard error.
